# Methylglyoxal-Induced Dysfunction in Brain Endothelial Cells via the Suppression of Akt/HIF-1α Pathway and Activation of Mitophagy Associated with Increased Reactive Oxygen Species

**DOI:** 10.3390/antiox9090820

**Published:** 2020-09-03

**Authors:** Donghyun Kim, Kyeong-A Kim, Jeong-Hyeon Kim, Eun-Hye Kim, Ok-Nam Bae

**Affiliations:** College of Pharmacy Institute of Pharmaceutical Science and Technology, Hanyang University, Ansan 15588, Korea; ssks7787@naver.com (D.K.); hosangkka@naver.com (K.-AK.); tha6318@naver.com (J.-H.K.); rladmsgp615@naver.com (E.-H.K.)

**Keywords:** methylglyoxal, hypoxia-inducible factor 1 (HIF-1α), mitophagy, oxidative stress, brain endothelial cells

## Abstract

Methylglyoxal (MG) is a dicarbonyl compound, the level of which is increased in the blood of diabetes patients. MG is reported to be involved in the development of cerebrovascular complications in diabetes, but the exact mechanisms need to be elucidated. Here, we investigated the possible roles of oxidative stress and mitophagy in MG-induced functional damage in brain endothelial cells (ECs). Treatment of MG significantly altered metabolic stress as observed by the oxygen-consumption rate and barrier-integrity as found in impaired trans-endothelial electrical resistance in brain ECs. The accumulation of MG adducts and the disturbance of the glyoxalase system, which are major detoxification enzymes of MG, occurred concurrently. Reactive oxygen species (ROS)-triggered oxidative damage was observed with increased mitochondrial ROS production and the suppressed Akt/hypoxia-inducible factor 1 alpha (HIF-1α) pathway. Along with the disturbance of mitochondrial bioenergetic function, parkin-1-mediated mitophagy was increased by MG. Treatment of N-acetyl cysteine significantly reversed mitochondrial damage and mitophagy. Notably, MG induced dysregulation of tight junction proteins including occludin, claudin-5, and zonula occluden-1 in brain ECs. Here, we propose that diabetic metabolite MG-associated oxidative stress may contribute to mitochondrial damage and autophagy in brain ECs, resulting in the dysregulation of tight junction proteins and the impairment of permeability.

## 1. Introduction

Vascular complications are the main causes of death in patients with diabetes [1]. There has been great interest in elucidating the mechanism underlying vascular dysfunction under diabetic conditions [2,3,4]. High glucose significantly increased the generation of reactive oxygen species (ROS) and apoptosis in endothelial cells (ECs) [4]. We have recently observed autophagy activation accompanying the increased intracellular ROS, mitochondrial dysfunction, and angiogenic impairment in high glucose exposed endothelial progenitor cells [3]. Notably, it has been suggested that methylglyoxal (MG), a dicarbonyl glycolysis by-product, may contribute to diabetic vascular endothelial damage. Higher plasma levels of MG have been observed in patients with diabetes than in non-diabetics [5,6,7]. MG can penetrate cells and react with arginine, lysine, and cysteine residues in proteins to form advanced glycation end products (AGEs). Previous studies have focused on several mechanisms, such as oxidative stress, inflammation, apoptosis, and involvement of AGE/the receptors for AGE (RAGE) signaling to explain MG-induced endothelial dysfunction [8,9,10,11].

Interestingly, hyperglycemia is one of the major risk factors for the development of cerebrovascular diseases [1]. Several studies have demonstrated the accumulation of ROS in high glucose- or MG-exposed brain ECs to elucidate the mechanisms underlying diabetic cerebrovascular impairment [12,13,14]. Brain ECs are functionally specialized cells composing the blood–brain barrier (BBB), which restricts the permeability of substances from the blood to the brain [15,16]. Several tight junction (TJ) proteins such as claudins, occludin, and zonular occludens (ZOs), tightly seal the junctions between adjacent brain ECs [17,18]. We have recently shown that impairment of TJ proteins by excessive autophagy may lead to ischemic EC damage, suggesting that the potential role of autophagy in BBB dysfunction [19,20].

Oxidative stress is known to play important roles in MG-induced endothelial damage [13,14,15]. The suppression of the antioxidant systems including hypoxia-inducible factor 1 (HIF-1) and the increase in mitochondrial ROS contribute to the enhancement of ROS formation [21,22]. Recent studies suggest that oxidative stress and mitochondrial damage associates BBB permeability alteration [23,24]. However, the role of oxidative stress and the crosstalk between mitochondrial damage and autophagy in MG-induced brain EC dysfunction has never been demonstrated.

In this study, we investigated the effects of MG on brain EC function and the underlying mechanisms of MG toxicity, including the imbalance of MG detoxification mechanisms, the suppression of adaptive responses, and the activation of mitochondrial oxidative stress and mitophagy associated with bioenergetics disturbance, which could ultimately affect TJ integrity and permeability impairment in brain ECs.

## 2. Materials and Methods

### 2.1. Materials

MG, 3-methyladenine (3-MA), N-acetyl-L-cysteine (NAC), bafilomycin A1 (Baf A), albumin from bovine serum (BSA), thiazolyl blue tetrazolium bromide (MTT), propidium iodide (PI), fluorescein isothiocyanate (FITC)-dextran, and other chemicals were purchased from Sigma Aldrich (St. Louis, MO, USA). MitoTracker Red CMXRos was purchased from Invitrogen (Burlington, ONT, Canada). FPS-ZM1, a specific antagonist for RAGE, was from Millipore (#553030; Temecula, CA, USA).

### 2.2. Cell Culture

Immortalized mouse brain endothelial cells (ECs), bEnd.3 cells (ATCC, Manassas, VA, USA), were cultured in Dulbecco’s modified Eagle’s medium (DMEM, 4500 mg/L D-glucose, L-glutamine, 110 mg/L sodium pyruvate, 1.5 g/L sodium bicarbonate; Welgene, Daegu, Korea) supplemented with 10% fetal bovine serum (FBS, Mediatech Inc., Manassas, VA, USA), 100 units/mL penicillin, and 100 μg/mL streptomycin (Welgene). bEnd.3 cells were incubated in a humidified incubator containing 5% CO_2_ at 37 °C until confluence.

### 2.3. PI Fluorscence and MTT Reduction Assay

The bEnd.3 cells were seeded on 96-well plates at a density of 5 × 10^3^ cells/well and incubated to confluence. To measure the non-viable bEnd.3 cells after MG exposure, PI (25 μg/mL), a fluorescent DNA-binding dye that freely penetrates cell membranes of dead or dying cells, was used. Cells treated with membrane lysis buffer (Promega, Madison, WI, USA) were used as a positive control. After 10 min of incubation, the fluorescence of PI was measured using an excitation and emission wavelength of 493 and 632 nm, respectively, in EnSpire multimode spectrophotometer (PerkinElmer, Santa Clara, CA, USA). To evaluate the MTT reduction potential of bEnd.3 cells after MG exposure, we added 0.5 mg/mL MTT at the end of the stimulus exposure [25]. After 2 h of incubation, formazan was dissolved in dimethyl sulfoxide. The absorbance was measured at 570 nm using EnSpire multimode spectrophotometer. In experiments with inhibitors including NAC, 3-MA or Baf A, inhibitors were pretreated for 1 h, and MG was added in the presence of inhibitors for 6 h.

### 2.4. In Vitro Permeability Assay

The bEnd.3 cells were seeded on a 6.5 mm Transwell^®^ with a 0.4-µm pore polycarbonate membrane insert (Corning, New York, NY, USA) at a density of 2 × 10^4^ cells/well and incubated for six days until confluence. Old media were replaced with fresh media every 2–3 days and the cells were treated with MG on day seven. Transendothelial electrical resistance (TEER) and fluorescence-based permeability assay were used to evaluate the effects of MG on the permeability of endothelial barriers. For the fluorescence-based permeability assay, 20 µg/mL of FITC-dextran (Sigma-Aldrich) was added to the upper chamber of the Transwell^®^, and fresh phosphate-buffered saline (PBS) was plated in the lower chamber. After 30 min of incubation (37 °C, 5% CO_2_), the fluorescence of FITC-dextran in the lower chamber was measured with an Enspire plate reader (PerkinElmer, Santa Clara, CA, USA) using an excitation and emission wavelength of 490 and 520 nm, respectively. TEER was measured immediately after MG treatment using an EVOM2 voltohmmeter and STX2 electrode (World Precision Instruments, Sarasota, FL, USA). Data were subtracted by the value of the cell-free inserts of each treatment group.

### 2.5. Immunofluorescence Staining

The bEND.3 cells were seeded on Lab-Tek™ 8-well chambered coverslips (Thermo Fisher Scientific, Rochester, NY, USA) at a density of 9 × 10^3^ and incubated to confluence. After MG treatment, cells were fixed and permeabilized with ice-cold methanol and acetone, respectively, blocked with 5% normal donkey serum (Sigma Aldrich) in PBS, and incubated for 1 h. The cells were then incubated with primary antibodies diluted in PBS containing 1% normal donkey serum before attaching fluorescent secondary antibodies diluted in 1% BSA for visualization. Fluorescence was recorded using a K1-Fluo confocal laser scanning microscope (Nanoscope Systems, Daejeon, South Korea), and the co-localization of the proteins was analyzed with the ImageJ plug-in JACoP (Just Another Co-localization Plugin).

### 2.6. Cellular ROS Detection Assay

To measure the total amount of cellular ROS, we used the 2’,7’-dichlorofluorescein diacetate (DCF-DA) cellular ROS detection assay kit (Abcam, Cambridge, MA, USA) according to the manufacturer’s instructions. Briefly, bEND.3 cells were seeded on 96-well plates or Lab-Tek™ 8-well chambered coverslips at the same density as described above and incubated to confluency. DCF-DA (25 μM) was added to the cells 30 min before MG treatment. After MG treatment, the fluorescence of DCF, which is the oxidized form of DCF-DA, was measured using an excitation and emission wavelength of 485 and 535 nm, respectively. Data were calculated as fold change compared to time point 0 in each group.

### 2.7. Mitochondrial ROS Detection Assay

MitoSOX Red mitochondrial superoxide indicator (Invitrogen) was used to visualize mitochondrial ROS in bEND.3 cells. bEND.3 cells were seeded and maintained on a Lab-Tek™ 8-well chambered coverglass as described above. At the end of treatment, the old medium was discarded, and the cells were incubated with MitoSOX (5 μM) at 37 °C for 10 min. The cells were then fixed in 2% paraformaldehyde and permeabilized with ice-cold acetone for counterstaining with 0.15 μg/mL 4′,6-diamidino-2-phenylindole (DAPI; Vector Laboratories, Burlingame, CA, USA). Fluorescence was recorded using a K1-Fluo confocal laser scanning microscope (Nanoscope Systems) and quantified with ImageJ.

### 2.8. Mitochondrial Fractionation

Mitochondrial fractions from vehicle- or MG-treated bEND.3 cells were prepared using the Mitochondria/Cytosol Fractionation kit (Biovision, Milpitas, CA, USA). Briefly, treated cells were collected, washed once with cold PBS, and centrifuged at 600× *g* for 5 min at 4 °C. Cells were then resuspended in ice-cold extraction buffer mix containing 1 mM DTT and protease inhibitor cocktail and briefly sonicated for lysis. Cell lysates were incubated on ice for 10 min and centrifuged at 700× *g* for 10 min at 4 °C. Supernatants were collected and centrifuged at 10,000× *g* for 30 min at 4 °C and separated into cytosol (supernatant) and mitochondria (pellet) fractions. Pellets were resuspended in extraction buffer and subjected to western blot analysis.

### 2.9. Measurement of Bioenergetic Function

**Scheme 1:**
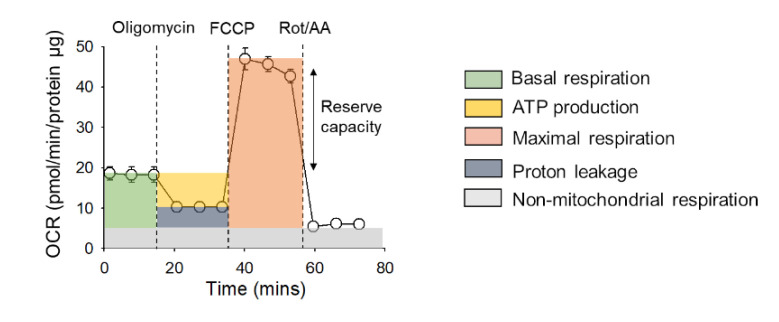
Calculation of cellular bioenergetic profile in bEND.3 cell

The bioenergetic function in bEND.3 cells was measured by using the XFp analyzer and Mitostress test kit (Agilent, Santa Clara, CA, USA) according to the manufacturer’s instructions. The real-time oxygen consumption rate (OCR) was monitored with the sequential injection of modulators for the mitochondrial electron transport chain (ETC). Briefly, bEND.3 cells were seeded on the XFp miniplate (Agilent) at a density of 1.6 × 10^5^ cells/well. At 6 h after MG treatment, the old medium was replaced to assay medium (DMEM, pH 7.4; Agilent) containing 5.5 mM glucose, 1 mM sodium pyruvate, and 2 mM L-glutamine. The cells were incubated for 1 h in a non-CO2 incubator to allow proper degassing before analysis. During analysis, oligomycin, carbonyl cyanide-4-(trifluoro-methoxy)phenylhydrazone (FCCP), and rotenone/antimycin A (Rot/AA) were sequentially injected at final concentrations of 2.5, 2.0, and 1.0 μM, respectively, through an XFp cartridge. Basal respiration, ATP production-linked oxygen consumption, maximal respiration, proton leakage, and non-mitochondrial respiration were calculated from the OCR profile, as shown in Scheme 1 below. OCR values were normalized by the protein amount of each well, which was measured by bicinchoninic acid-protein assay (Pierce™ BCA Protein Assay Kit; Thermo Fisher Scientific, Rockford, IL, USA) [26]. In experiments with NAC, NAC (5 mM) were pretreated for 1 h, and MG was added in the presence of NAC for 6 h.

### 2.10. Mitochondrial Mass Analysis

To measure the mitochondrial mass, we used nonyl acridine orange (NAO; Molecular Probes, Eugene, OR, USA) which is a fluorescent probe that accumulates in the mitochondria [27]. The bEND.3 cells were seeded on 96-well plates as described above. At the end of treatment, 1 μM NAO was added to the cells. After 15 min of incubation, the fluorescence of NAO was measured using an excitation and emission wavelength of 490 and 520 nm, respectively, in EnSpire multimode spectrophotometer. The fluorescence intensity was normalized by protein amounts of each well.

### 2.11. Western Blot Analysis

Western blot analysis was performed as previously described [19]. Primary antibodies against occludin (c-term), ZO-1, and claudin-5 and secondary antibodies, Alexa Fluor 488 donkey anti-mouse and Alexa Fluor 555 donkey anti-rabbit, were purchased from Thermo Fisher Scientific. To detect the formation of AGE products with MG-modification, mouse anti-methylglyoxal monoclonal antibodies (STA-011; Cell Biolabs, San Diego, CA, USA) were used. Primary antibodies against p-mTOR, t-Akt, p-Akt, t-S6K1, p-S6K1, HIF-1α, COXIV, VDAC, and Parkin (Prk8) were purchased from Cell Signaling Technology (4695S, Danvers, MA, USA). Primary antibodies against t-mTOR and occludin (E-5) and the secondary antibody donkey anti-rabbit IgG-FITC were purchased from Santa Cruz Biotechnology (Dallas, TX, USA). The primary antibody against LC3B was purchased from Sigma Aldrich. Primary antibodies against Glo-1 and Glo-2 were purchased from Abcam. To counterstain the total protein in the gel, Coomassie Brilliant Blue staining was used as previously described [8].

### 2.12. Statistical Analysis

All experimental values were expressed as the mean and standard error (SEM). Statistical significance between groups was determined by the Student’s t-test and one-way analysis of variance (ANOVA) followed by Tukey’s post hoc test. SPSS Version 24 was used for data analysis. In all analyses, a *p* value < 0.05 was considered statistically significant.

## 3. Results

### 3.1. MG induced Accumulation of MG-Adducts and Deterioration of the Glyoxalase System in bEND.3 Cells

**Figure 1:**
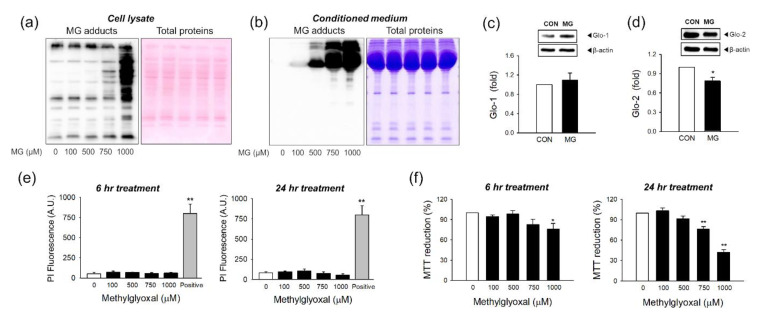
Formation of Methylglyoxal (MG)-adducts and alteration of cell viability in bEnd.3 cells after MG treatment. (**a**–**d**) bEnd.3 cells were incubated with MG at different concentrations (0–1000 μM) for 24 h. MG-adduct formation in (**a**) the cell lysate or (**b**) the conditioned medium was determined by western blotting and Coomassie Brilliant Blue staining. Representative images were shown. The expression levels of (**c**) glyoxalase (Glo)-1 and (**d**) Glo-2 were examined after cell treatment with 1000 μM MG for 24 h (*n* = 3~4). (**e**) The changes in cell viability after MG exposure (6 h or 24 h) were determined by (**e**) PI staining or (**f**) MTT assay (*n* = 3). Data are presented as the mean ± SEM. * *p* < 0.05, ** *p* < 0.01 vs. control (CON).

MG is a potent glycating agent that induces the formation of advanced glycation end (AGE) products in proteins. Accumulating evidence has shown that these products increase vascular complications of diabetes [28]. To evaluate the effects of MG in the brain endothelial bEND.3 cell line, the formation of MG adducts in the cell lysate and conditioned medium was investigated following incubation of MG (0–1000 μM) for 24 h. MG adducts were increased in both the cell lysate and conditioned in an MG concentration-dependent manner (Figure 1a,b). To investigate the effects of MG detoxification systems, we measured Glo-1 and -2 enzyme levels. After 24 h of MG treatment, there was a slight increase in the Glo-1 expression level compared to that in the vehicle control, but this increase was not statistically significant (Figure 1c). In contrast, Glo-2 expression was significantly decreased, and the expression level was approximately 79% of that in the vehicle control after 24 h of MG treatment (Figure 1d).

We investigated if MG affected cell viability in bEND.3 cells using two different methods. Staining with propidium iodide (PI), a fluorescent dye which freely penetrates dead or dying cells to differentiate these cells from living cells [29], was not increased in brain ECs either 6 h or 24 h after MG treatment (Figure 1e). On the contrary, MG significantly decreased the extent of MTT reduction to formazan, which is proportional to the number of metabolically viable cells [29], in bEND.3 cells in treatment time- and concentration-dependent manner (Figure 1f).

### 3.2. Mitochondrial and Total Cellular ROS Production Increases after MG Exposure with the Suppression of the Akt/HIF-1α Pathway

**Figure 2:**
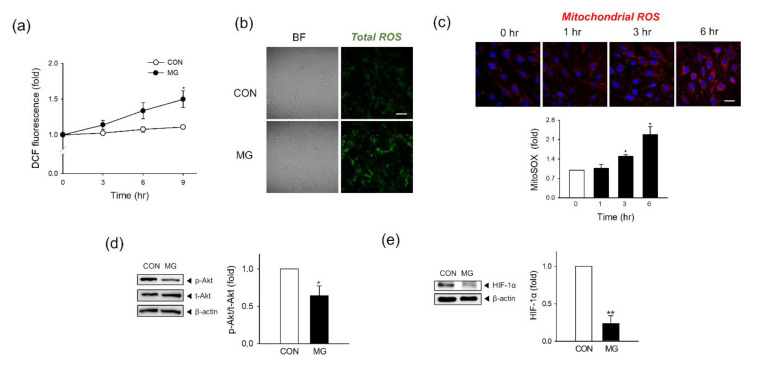
Total cellular and mitochondrial reactive oxygen species (ROS) production and suppression of the Akt/HIF-1α pathway after MG exposure. (**a**) Total ROS formation was detected in DCF-DA-stained bEND.3 cells at the indicated times (*n* = 3). (**b**) Confocal microscopy images of DCF-DA-stained bEND.3 cells 24 h after MG treatment. Scale bar: 20 μm. (*n* = 3). (**c**) Mitochondrial ROS formation was measured at 0, 1, 3, and 6 h after MG treatment by confocal microscopy. Scale bar: 20 μm. Fluorescence was quantified from three independent experiments. (*n* = 3). Representative images are shown. (**d**,**e**) The protein levels of (**d**) phosphorylated and total Akt (p-Akt and t-Akt) and (**e**) HIF-1α were determined by western blotting. Protein levels were normalized to β-actin levels (*n* = 3). Data are presented as the mean ± SEM. * *p* < 0.05, ** *p* < 0.01 vs. control (CON).

Next, we measured MG-induced ROS formation at the mitochondrial and total cellular levels. As measured by DCF fluorescence in fluorimeter, total cellular ROS formation gradually increased with MG exposure time, and the difference relative to the control became significant 9 h after MG treatment (Figure 2a). This result matched well to the fluorescence images showing an increased ROS level in the MG-treated cells for 24 h compared to those in the control group (Figure 2b). To detect mitochondrial ROS, we stained cells with MitoSOX Red mitochondrial superoxide indicators after MG treatment. Red fluorescence was observed under a confocal laser scanning microscope, and cell count-based normalization was used to analyze the fluorescence intensities. Mitochondrial ROS increased over time, and the difference relative to time point 0 was significant 3 h after treatment (Figure 2c). At 24 h post-treatment, the mitochondrial ROS production in MG-treated cells was higher than that in vehicle-treated cells (Supplementary Figure S1).

Hypoxia-inducible factor 1α (HIF-1α) is known to control the oxygen demand through the mitochondria affecting cellular and mitochondrial ROS status [30], and the expression of HIF-1 is regulated by the phosphorylation of protein kinase B (Akt or PKB) [31]. After 1 h of MG exposure (1000 μM), the phosphorylation of Akt in brain ECs was significantly decreased by approximately 36% compared to that in the vehicle control (Figure 2d). HIF-1 α expression was temporarily increased at 1 h post-treatment but decreased afterward with time (Supplementary Figure S2). At 24 h MG exposure (1000 μM), HIF-1α expression was significantly decreased by approximately 77% compared to that in the vehicle control (Figure 2e).

### 3.3. Disturbnace of Bioenergetic Mitochondrial Function and Activates Parkin-Mediated Mitophagy by MG in Brain ECs

**Figure 3:**
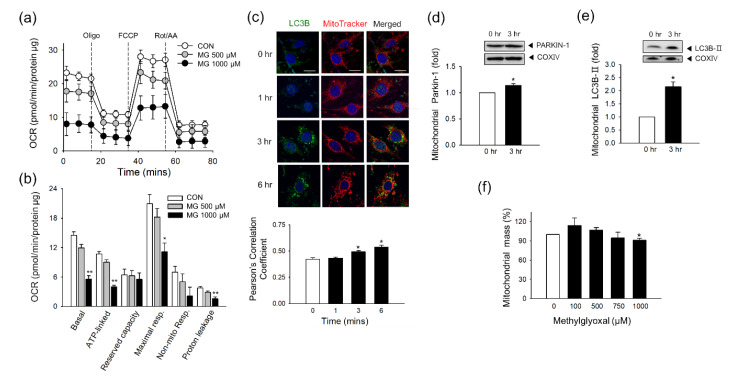
Mitochondrial bioenergetic disturbance and activation of mitochondrial autophagy following MG treatment in brain endothelial cells (ECs). (**a**,**b**) bEnd.3 cells were incubated with MG for 6 h and applied to Seahorse MitoStress Assay (*n* = 3). (**a**) The profile of the oxygen consumption rate was plotted. (**b**) The parameters for mitochondrial respiration were calculated. (**c**) The localization of LC3B and mitochondria was examined at indicated times after MG treatment by confocal microscopy. Mitochondria were labeled by MitoTracker. Pearson’s correlation coefficients were calculated from three independent experiments (*n* = 3). Representative images are shown. Scale bar: 20 μm. (**d**,**e**) The protein level of (**d**) Parkin-1 and (**e**) LC3B-II in the mitochondrial fraction was determined by western blotting. Protein levels were normalized to that of cytochrome C oxidase subunit 4 (COXIV) (*n* = 3). (**f**) Mitochondrial mass was determined by staining with NAO (*n* = 3). Data are presented as the mean ± SEM. * *p* < 0.05, ** *p* < 0.01 vs. control (CON).

Brain ECs have a very high-energy demand, and mitochondrial bioenergetic function is critical to maintaining their unique and distinct barrier function [26]. We observed that MG increased the generation of total and mitochondrial ROS along with the suppression of defense mechanisms of p-Akt/HIF-1. Therefore, we next investigated if MG treatment disturbs mitochondrial function using a measurement of oxygen consumption rate. The basal respiration and respiration after the sequential addition of oligomycin (Oligo), FCCP, and combination of rotenone and antimycin A (Rot/AA) were monitored. As shown in Figure 3a, the mitochondrial respiration of brain ECs was decreased by 6 h incubation of MG. Basal respiration, ATP-linked respiration, proton leakage, and maximal respiration were decreased by MG with a statistical significance (Figure 3b). The maximal respiratory capacity, calculated from the oxygen consumption after FCCP stimulation, is known to be a strong indicator of the potential of mitochondrial energetic dysfunction [32].

Accumulating evidence indicates that mitophagy is activated as an adaptive response to oxidative stress and mitochondria damage [33,34,35], and we investigated the activation of mitophagy in MG-exposed brain ECs. The formation of autophagosomes was increased following MG treatment, as measured by immunostaining of microtubule-associated proteins 1B light chain 3B (LC3B). The autophagosomes were co-localized with mitochondria (Figure 3c), and the increased mitophagy was continued at 24 h after MG stimulation (Supplementary Figure S3). Since Parkin-1 is involved in the clearance of damaged mitochondria through LC3B recruitment [20,36], the protein level of Parkin-1 or LC3B-II was investigated in isolated mitochondria. At 3 h post-treatment of MG, Parkin-1 or LC3B-II level was increased by approximately 1.13- and 2.29-fold, respectively, in the mitochondrial fraction of brain ECs (Figure 3d,e). Next, we measured if mitochondrial mass in bEnd.3 was affected by MG treatment for 24 h using nonyl acridine orange (NAO). While there is a statistical significance, the mitochondrial mass decreased only by 8.98% in 1000 μM of MG-treated cells, compared to the control cells (Figure 3f).

### 3.4. Restoration of Mitochondrial Function and Mitophagy by NAC

**Figure 4:**
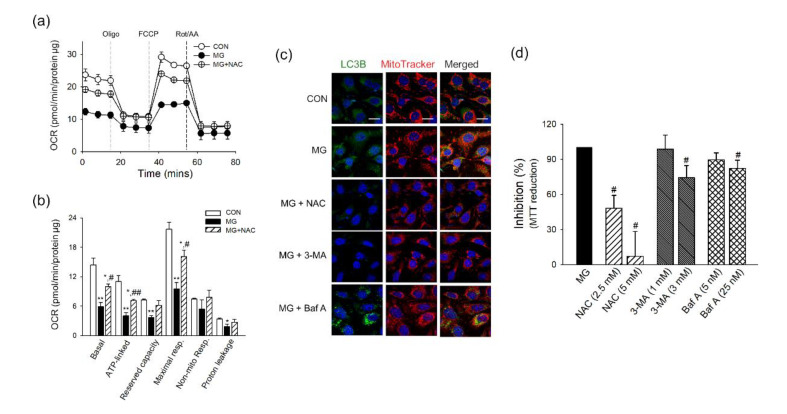
Effects of NAC and autophagy inhibitors on mitochondrial damage induced by MG in brain ECs. (**a**,**b**) bEnd.3 cells were treated with NAC (5 mM) for 1 h before and during MG stimulation (1000 μM) for 6 h. Cells were applied to Seahorse MitoStress Assay (*n* = 3). (**a**) The profile of the oxygen consumption rate was plotted. (**b**) The parameters for mitochondrial respiration were calculated. (**c**,**d**) NAC or autophagy inhibitors (3-MA or Baf A) were treated for 1 h before MG treatment, and maintained for 6 h during MG treatment. (**c**) The localization of LC3B and mitochondria was examined by confocal microscopy. Mitochondria were labeled by MitoTracker. Representative images are shown. Scale bar: 20 μm. (**d**) The effects of NAC or autophagy inhibitors (3-MA or Baf A) on MG-attenuated MTT reduction were determined (*n* = 3~4). Data are presented as the mean ± SEM. * *p* < 0.05, ** *p* < 0.01 vs. control (CON). # *p* < 0.05, ## *p* < 0.01 vs. MG-treated cells.

To examine the role of oxidative stress in mitochondrial damage by MG, we treated brain ECs with NAC (5 mM), a well-established antioxidant, for 1 h before and during MG exposure (6 h). As shown in Figure 4a, NAC pretreatment significantly reversed MG-induced mitochondrial energetic impairment. Basal respiration, ATP-linked respiration, and maximal respiration, which were significantly impaired by MG, were significantly restored by NAC treatment (Figure 4b). Next, we examined if NAC or autophagic inhibitors, 3-MA or Baf A, affects MG-induced mitophagy. The co-localization of LC3B and MitoTracker, an indicator for the formation of autophagosomes containing mitochondria, was increased by MG in brain ECs, and the treatment of NAC or 3-MA reversed the co-localization suggesting the mitophagy was inhibited by NAC or 3-MA (3 mM) (Figure 4c). Meanwhile, the restoring effect of Baf A (25 nM) on the co-localization of LC3B and MitoTracker was not found. Baf A is known to block late steps of autophagy after autophagosome formation by inhibiting fusion between autophagosomes and lysosomes [37]. The significant inhibition by NAC was also observed in MG-attenuated MTT reduction, which is an indicator of metabolic impairment [29,38]. The 3-MA and Baf A also demonstrated a statistically significant inhibition against the MG effect on MTT reduction but to a lesser extent compared to NAC (Figure 4d).

### 3.5. Activated Autophagy Might Contribute to Occludin Degradation

**Figure 5:**
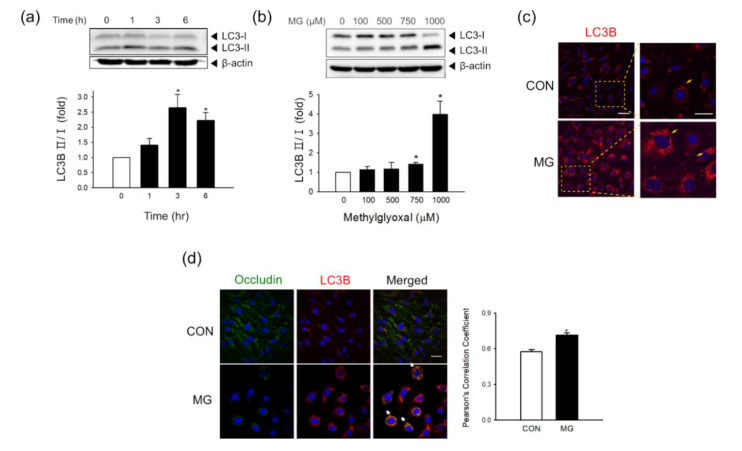
Autophagy activity and co-localization of occludin with LC3B 24 h after MG exposure in brain ECs. (**a**,**b**) The protein levels of LC3B-I/II were determined (**a**) at 0, 1, 3, or 6 h after treatment with 1000 μM MG (*n* = 3) or (**b**) at 24 h after treatment with 0–1000 μM MG by western blotting (*n* = 4). (**c**) MG-exposed bEnd.3 cells were stained with an antibody against LC3B and visualized by confocal microscopy. Scale bar: 50 μm. (*n* = 4) (**d**) Co-localization of occludin with LC3B was analyzed after MG exposure using confocal microscopy. Scale bar: 20 μm. Pearson’s correlation coefficients were calculated from three independent experiments (*n* = 3). Representative images are shown. Data are presented as mean ± SEM. * *p* < 0.05 vs. control (CON).

We recently demonstrated that autophagy contributed to occludin degradation in bEND.3 cells under hypoxic conditions [19]. To examine if autophagy occurs in brain ECs following MG stimulation, the total level of LC3B-I/II conversion was determined. Along with the enhanced mitophagy as observed in Figure 3, the conversion of LC3B-I/II was significantly increased after MG treatment in a time- and concentration-dependent manner (Figure 5a,b). To identify the involvement of autophagy in occludin degradation, we investigated the correlation between autophagy activation and occludin degradation at 24 h after MG treatment. An increase in autophagic puncta was found after treatment with 1000 μM MG, as observed by LC3B immunostaining (Figure 5c). Besides, we observed a decrease in occludin signals and a concurrent increase in the co-localization of LC3B with occludin after MG treatment in bEnd.3 cells (Figure 5d).

### 3.6. MG Induces Endothelial Dysfunction and Degradation of Tight Junction Proteins in bEND.3 Cells

**Figure 6:**
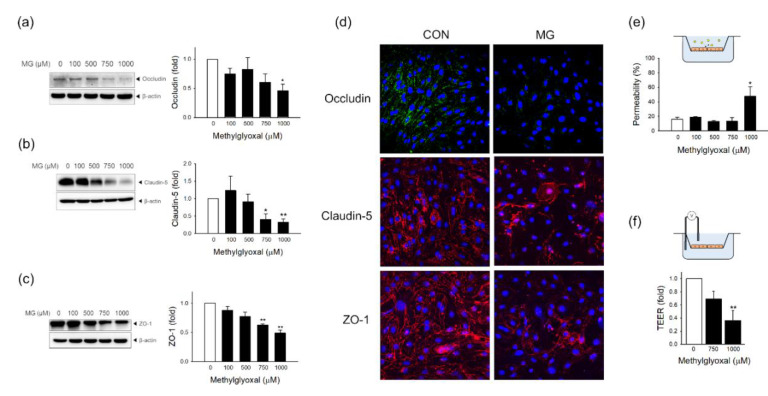
MG induces endothelial dysfunction through tight junction degradation and permeability destruction in bEND.3 cells. The protein expression levels of (**a**) occludin (*n* = 5), (**b**) claudin-5 (*n* = 4), and (**c**) ZO-1 (*n* = 3) were determined 24 h after treatment with 100–1000 μM MG by western blotting. (**d**) MG-exposed bEnd.3 cells were stained with an antibody against occludin, claudin-5, or ZO-1 and visualized by confocal microscopy. Scale bar: 50 μm. (**e**,**f**) Functional changes in endothelial permeability were measured by (**e**) an in vitro FITC-dextran permeability assay (*n* = 3–7) and (**f**) TEER measurements (*n* = 4) after MG treatment. Representative images are shown. Data are presented as mean ± SEM. * *p* < 0.05, ** *p* < 0.01 vs. control (CON).

To investigate the effects of MG on brain EC tight junction (TJ) proteins, which restrict the permeability of the brain-blood barrier, we measured the protein level of TJ proteins and the functional integrity of TJ. Western blot analysis showed that occludin was significantly reduced after treatment with 1000 μM MG (Figure 6a). The protein levels of claudin-5 and zonula occludens-1 (ZO-1) were significantly reduced after treatment with 750 and 1000 μM MG (Figure 6b,c). The integrity of TJ was disrupted by MG treatment, as found in the decreased fluorescence level and impaired distribution of these TJ proteins from the plasma membrane outlining (Figure 6d). Next, we investigated the change of functional integrity in brain ECs after MG treatment. We measured the permeability of FITC-conjugated dextran and TEER in MG-exposed ECs. The FITC-dextran permeability assay showed that MG at a concentration of 1000 µM significantly reduced the integrity of brain ECs (Figure 6e). TEER also significantly increased after treatment with 1000 μM MG (Figure 6f). These data demonstrate that MG induced endothelial dysfunction in bEND.3 cells, resulting in the disruption of permeability as a specialized barrier.

## 4. Discussion

In this study, we demonstrated that MG, an endogenous toxic metabolite under diabetic condition, induced dysfunction in brain endothelial bEND.3 cells. MG induced downregulation of Glo-2, which may disrupt MG detoxification mechanisms. Mitochondrial and total cellular ROS formation increased from the early stage of MG treatment. Akt was suppressed shortly after MG exposure, which might have affected the expression of HIF-1α. Mitochondrial energetic function was impaired by MG. Mitophagy and autophagy were activated, and autophagosomes were co-localized with occludin after MG treatment. TJ proteins including occludin, claudin-5, and ZO-1 were decreased in level and re-distributed from the plasma membrane leading to impairment of permeability of brain ECs following MG exposure. Since vascular dysregulation is one of the most severe complications associated with diabetes, there have been several attempts to elucidate the effect of MG on endothelial dysfunction [8,9,10,11]. We believe that our current observations contribute to an integrated understanding of the role of MG in diabetic endothelial impairment, especially in cerebrovascular dysfunction under diabetic condition.

Glyoxalase (Glo) is the major detoxification enzyme of MG, and up to 99% of MGO is detoxified by the glyoxalase system under physiological conditions [39]. Since the MG degradation process consists of two steps involving Glo-1 and -2, the expression level of these two enzymes is crucial for the whole MG detoxification mechanism [40]. Accumulating evidence has shown that the impairment of the glyoxalase systems is correlated with various complications of diabetes, including macrovascular disease in humans [41]. In this study, we observed that the intracellular accumulation of MG adducts was considerably increased at an MG concentration of 1000 μM. There was a slight but not significant increase in the Glo-1 level and a significant decrease in the Glo-2 level under the same conditions. Interestingly, Zhang et al. [35] demonstrated that HIF-1 negatively regulated the gene expression of Glo-1 in leukemia stem cells. Dafre et al. [33] demonstrated that autophagy inhibitors (bafilomycin A and chloroquine) reversed Glo-2 degradation induced by MG. Since we have shown that MG treatment induces HIF-1 degradation and autophagy activation, these mechanisms might affect the expression levels of Glo-1 and Glo-2 in ECs. However, this should be further investigated in future studies.

Previous studies have shown that ROS formation may be the crucial mechanism for MG cytotoxicity in endothelial cells (ECs) [42]. The cytotoxicity of MG was decreased by several compounds that reduce ROS formation in ECs [43,44,45]. While ROS has been suggested to be a key mediator for MG-induced EC damage, the role of mitochondrial oxidative stress or mitophagy has not been studied. Interestingly, recent studies have shown that mitochondrial ROS contribute to the MG toxicity in other tissue damage associated with diabetes. A recent report by Liu et al. [46] demonstrated that MG-induced mitochondrial ROS in rat pancreatic beta cells. Mitochondrial ROS production is increased when mitochondrial ETC systems are defective, leading to the upregulation of pathological mechanisms contributing to MG-induced tissue damage [22]. Here we report that mitochondrial ROS, bioenergetic disturbance, and mitophagy may play critical roles in dysfunction in brain ECs. Of note, the treatment of a representative antioxidant, NAC, significantly reversed MG-induced bioenergetic impairment and mitophagy, supporting the notion that ROS is the key mechanism for mitochondrial damage in brain ECs. In addition to mitochondrial ROS, non-mitochondrial sources such as eNOS uncoupling, iNOS activation, microsomal monooxygenases redox cycling, or lipid peroxidation products would need to be further examined to understand more comprehensively the role and impact of increased oxidative stress in brain ECs by MG [23,47].

Mitochondria are organelles that play an important role not only in energy production but also in maintaining endothelial functions by regulating signal transduction against environmental changes [48]. For example, in response to oxygen deprivation, the number of mitochondria can be adjusted to reduce the oxygen demand of the cells [49]. Accumulating evidence has shown that HIF-1 is the key regulator not only in adaptive responses to hypoxia but also in attenuating oxidative stress by regulating the number of mitochondria [30]. HIF-1 activates the autophagy of mitochondria to remove damaged mitochondria when oxidative stress is increased [50]. When these adaptive responses fail, damaged mitochondria accumulate, and electron leakage from the ETC systems may amplify ROS formation. Bento et al. [51] showed that, during hypoxia, HIF-1α expression was decreased in retinal pigment epithelial cells after MG treatment. In the current study, we have shown that the alteration of Akt/HIF-1 α, mitochondrial ROS generation, mitochondrial energetic impairment, and mitophagy in MG-exposed brain ECs, suggesting that these interrelated phenomena simultaneously contribute to the MG-induced functional disruption in permeability change in brain ECs.

In this study, using mitochondrial superoxide indicators, we observed increased mitochondrial ROS levels in bEnd.3 cells at the early stages of MG exposure and until 24 h. Total cellular ROS formation was concurrently increased with mitochondrial ROS. Furthermore, we showed that autophagy of mitochondria was activated during MG-induced ROS formation and that HIF-1α expression was temporarily increased 1 h after MG treatment, which might play a role in the activation of mitochondrial autophagy. ROS-mediated mitochondrial functional damage, as observed in bioenergetic disturbance, increased after MG treatment, and these damaged mitochondria may activate mitophagy. We tried to elucidate if mitochondrial mass, which represents the changes in mitochondrial content [52], is affected by MG treatment using a specific dye of nonyl acridine orange [27]. Despite a statistical significance, we could only observe a very small reduction in mitochondrial mass (Figure 3f). Considering that the mitochondrial dysfunction in type I and type II diabetic patients has been clinically emphasized in a recent few years [53,54], further studies are warranted to study the effect of MG on mitochondrial dynamics, including fusion, fission, and mitophagy [55]. Growing evidence has demonstrated that mitochondrial dysfunction associated with ROS in the BBB is critical in BBB alteration in pathological conditions of vascular and neurological diseases [23]. Considering that uncontrollable or inadequate mitophagy can have detrimental effects on cells [56], studies to elucidate how MG-associated mitophagy affects molecular mechanisms in BBB disruption should be further expanded.

Hypoxia-inducible factor 1 (HIF-1) is an intracellular oxygen sensor that reduces hypoxic damage in cells by reducing the oxygen demand through the suppression of mitochondrial biogenesis and activation of mitophagy [30]. Transcription, translation, and stability of HIF-1 can be regulated by post-translational phosphorylation by the Akt/mTOR/S6k1 pathway [31]. We showed that the activation of Akt was suppressed at 1 h post-treatment. Considering the role of Akt in the upregulation of the HIF-1α expression level [57], it may affect the low HIF-1α level after MG exposure. Degradation mechanisms reported by Bento et al. [51] may also be involved in the decrease of HIF-1α expression, but additional studies are needed. Here we demonstrated that modulation of HIF-1α might be involved in MG-induced ROS formation and functional impairment in brain ECs, and HIF-1α modulation may be a preventive strategy in patients with diabetes to reduce cardiovascular complications. This is in line with recent opinions that HIF prolyl hydroxylase (PHD) inhibitors may have a therapeutic potential in neurological diseases such as stroke by modulating HIF and ROS [58].

We observed the accumulation of intracellular or released protein MG-adducts (Figure 1a,b). MG produces AGEs by the binding to the arginine residue of the protein, and the most common MG-derived AGEs form is MG-derived hydroimidazolone-1 (MG-H1), and this can be particularly recognized by the receptors for AGEs (RAGE) mediating AGE toxicity [59]. We recently demonstrated that MG induced angiogenic impairment in EPCs via the AGE/RAGE-VEGFR-2 pathway [8]. However, when using FPS-ZM1, a RAGE antagonist, we could not observe any changes of MG effect on the impaired metabolic capacity of MTT reduction (Supplementary Figure S4a). The protein level of RAGE in brain ECs was not affected by MG treatment (Figure S4b). The role of the AGE/RAGE pathway is not likely to be actively involved at least in metabolic perturbation induced by MG in brain ECs; however, other potential effects on BBB function such as membrane TJ dynamics could not be excluded.

Our current study has significant limitations, especially in terms of the lack of clinical or translational relevance of the concentration of MG used in our experimental system. Previous clinical observations have demonstrated that the level of MG significantly increased in patients with diabetes [7,60]. Normal human plasma levels of MG are in the range of 50–300 nM, and higher plasma levels of MG are found for example, in patients with diabetic neuropathy, as high as 600–900 nM [61]. Wang et al. [7] reported that the range of plasma MG concentration could increase up to 5.9 ± 0.7 μM in patients with diabetes compared to up to 3.3 ± 0.4 μM in non-diabetic subjects. Fleming et al. [62] has shown the intracellular level of MG in erythrocytes as up to 25-fold higher in patients with diabetes compared to individuals without diabetes. The concentration of MG used in this study was up to 1000 μM, which is approximately at least 200-fold higher than the plasma concentration observed in patients with diabetes. This could be partly justified by a limited in vitro experimental condition with relatively short-time exposure. While vascular tissues under clinical diabetic conditions are chronically exposed to MG for years, cultured brain ECs might be temporarily exposed to MG, i.e., for up to 24 h in our case. This has been a limiting factor for studies on MG toxicity in cells, and similar ranges of MG concentrations applied in this study were also used in previous in vitro studies [63,64]. Nevertheless, we hope that our current observation could give insight into the BBB damage observed in diabetes, in terms of mechanistically valid MG effects on ROS generation, mitochondrial damage/mitophagy and barrier function alteration in a concentration and time-dependent manner in ranges without cell death as observed by PI fluorescence (Figure 1e). Further studies with clinical observations for MG-associated mitochondrial bioenergetic dysfunction or mitophagy in BBB would be interesting. Considering that BBB damage can be induced by endogenous stimuli such as hypoxia [20] or exogenous chemicals such as environmental pollutants [18], future studies regarding synergistic effects of the lower concentration of MG on these effects would also be necessary.

Growing evidence has shown that basal autophagy is essential to maintain proper vascular function [65], but at the same time, endothelial autophagy showed a detrimental role in the pathogenesis of vascular diseases [20]. In a recent study, we explored the role of autophagy during ischemic stress in brain ECs and showed that autophagy promotes endothelial dysfunction through occludin degradation [19]. In the case of MG-related autophagy, Fang et al. [66] showed that autophagy was activated at an early stage (1–6 h) of MG exposure in human brain microvascular ECs. In the same study, diabetic rats showed increased permeability of brain ECs after permanent middle cerebral artery occlusion, which means that brain ECs were more susceptible to ischemic stimuli under diabetic conditions. While NAC, an antioxidant, significantly reversed both mitochondrial metabolic dysfunction and mitophagy, 3-MA or Baf A, autophagy inhibitors, failed to reverse the metabolic impairment. This suggests that oxidative stress might be a causative factor leading to mitochondrial energetic disturbance and consequent mitophagy. In this study, we demonstrated that activated autophagy is involved in the removal of damaged mitochondria. These results indicate a dual role for autophagy in MG-exposed ECs, and the future studies exploring the contribution of MG-associated autophagy activation in terms of the different time window and the extent of autophagic activation.

## 5. Conclusions

This study demonstrated that MG-induced endothelial dysfunction through ROS production and increased mitochondrial damage, with high susceptibility to oxidative stress, contributes to the amplification of MG toxicity. Suppressed Akt expression might have a role in the insufficient response of HIF-1α to reduce oxidative damage. We also demonstrated the bioenergetic disturbance and activation of mitophagy after MG exposure. Tight junction proteins were decreased and re-localized, leading to impaired permeability in brain ECs.

## Supplementary Materials

The following are available online at https://www.mdpi.com/2076-3921/9/9/820/s1, Figure S1: Increased mitochondrial ROS in bEND.3 cells at 24 h after MG treatment., Figure S2: Alteration of HIF-1α protein level after MG treatment., Figure S3: Co-localization of mitochondria and autophagosomes in bEND.3 cells at 24 h after MG treatment. Figure S4. Involvement of RAGE in MG-induced changes in bEND.3 cells.
