# Supplementary material for: Methylglyoxal-Induced Dysfunction in Brain Endothelial Cells via the Suppression of Akt/HIF-1α Pathway and Activation of Mitophagy Associated with Increased Reactive Oxygen Species
Source: Antioxidants (Basel). 2020 Sep 3;9(9):820. doi: 10.3390/antiox9090820 (PMC7554889; doi:10.3390/antiox9090820)
Supplement: Supplementary file 1 [file antioxidants-09-00820-s001.pdf]

Supplementary Material

Methylglyoxal-induced dysfunction in brain endothelial cells via the suppression of Akt/HIF-1α pathway and activation of mitophagy associated with increased reactive oxygen species

Donghyun Kim [1,a], Kyeong-A Kim [1,a], Jeong-Hyeon Kim [1], Eun-Hye Kim [1] and Ok-Nam Bae [1,*]

[1] College of Pharmacy Institute of Pharmaceutical Science and Technology, Hanyang University, Ansan, Republic of Korea; ssks7787@naver.com (D.K.); hosangkka@naver.com (K.-A.K.); tha6318@naver.com (J.-H.K.); rladmsgp615@naver.com (E.-H.K.)

[a] These authors contributed equally to this study

* Correspondence: onbae@hanyang.ac.kr; Tel.: +82-31-400-5805 (O.-N.B.)

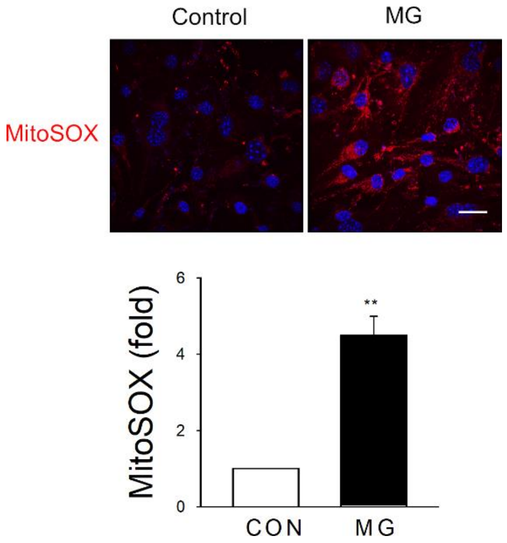

Figure S1. Increased mitochondrial ROS in bEND.3 cells at 24 hr after MG treatment. Mitochondrial ROS formation, as detected by MitoSOX, was monitored at the 24 hr after MG treatment using confocal microscopy. Scale bar: 20 μm. The fluorescence was quantified from three independent experiments (N = 3). Data are presented as mean ± SEM. **p<0.01 vs. CON.

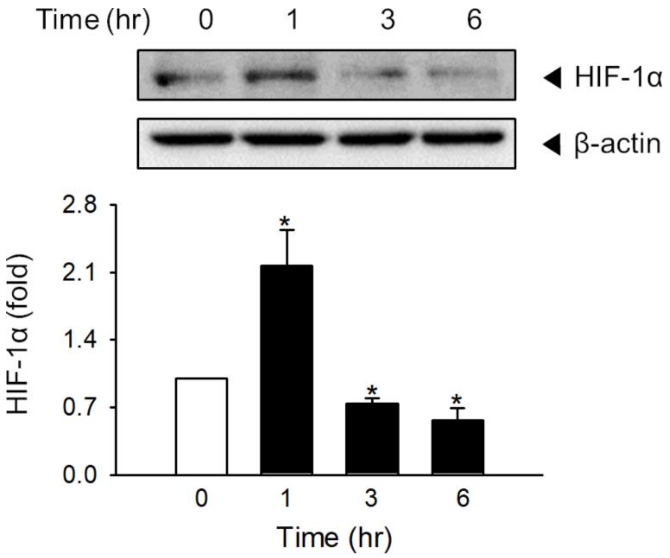

Figure S2. Alteration of HIF-1α protein level after MG treatment. HIF-1α was temporarily increased at 1 hr and then decreased after MG treatments. The protein levels of HIF-1α were determined by western blot. Protein levels were normalized by β-actin levels. N = 3. Data are presented as mean ± SEM. *$p<0.05$ vs. CON.

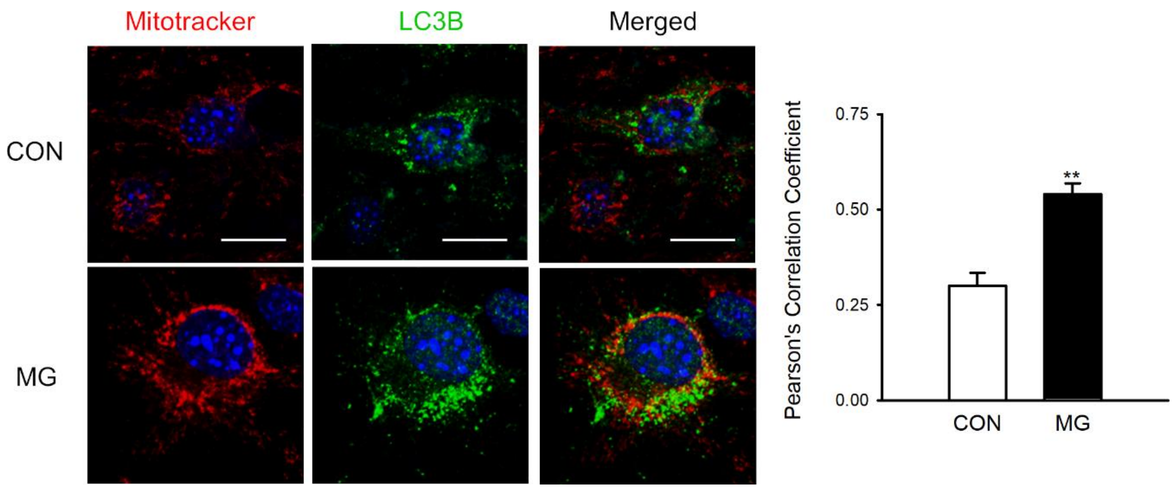

Figure S3. Co-localization of mitochondria and autophagosomes in bEND.3 cells at 24 hr after MG treatment. Localization of LC3B and mitochondria was examined at 24 hr after MG treatment by confocal microscopy. Pearson's correlation coefficients were calculated from the three independent experiments (N = 3). Scale bar: 20 μm. Data are presented as mean ± SEM. **p<0.01 vs. CON.

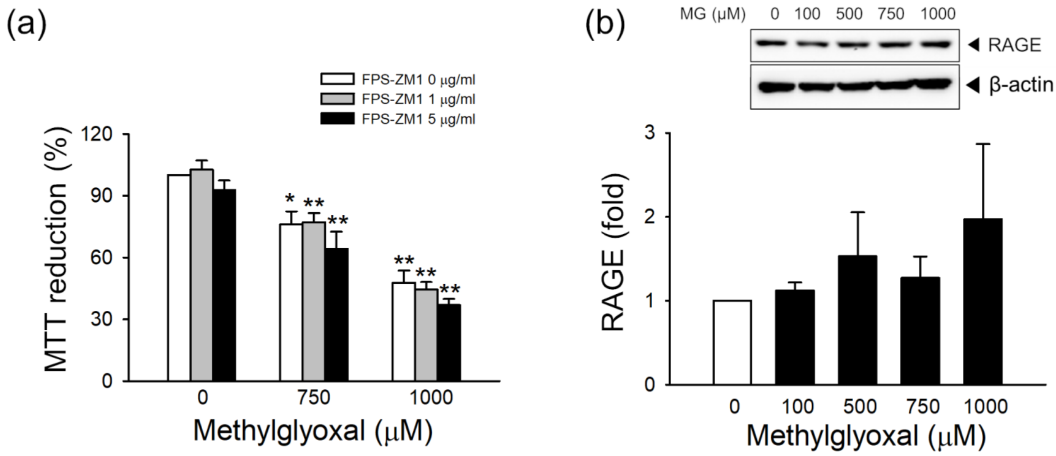

Figure S4. Involvement of RAGE in MG-induced changes in bEND.3 cells. (a) The cells were pre-treated with FPS-ZM1, a RAGE antagonist, for 4 h and then co-treated with MG at concentration of 0-1000 μM for 24 h. The extent of MTT reduction was determined (N = 4). (b) The protein level of RAGE in brain ECs were determined by western blotting (N = 3). Data are presented as mean ± SEM. *$p<0.05$, **$p<0.01$ vs. CON.
